# Characterization of protein biomarkers in Alzheimer's disease and dementia with Lewy bodies

**DOI:** 10.1002/alz70856_098343

**Published:** 2025-12-24

**Authors:** Aya Abdallah, Robert Allen, Dominik Domanski, Andrew M Goldfine, Sanjay Kumar, Laura Parkkinen, Becky C. Carlyle

**Affiliations:** ^1^ University of Oxford, Oxford, Oxfordshire, United Kingdom; ^2^ GSK, Collegeville, PA, USA

## Abstract

**Background:**

Alzheimer's Disease (AD) is characterized by amyloid‐beta‐positive neuritic plaques and tau‐positive neurofibrillary tangles, driven by factors like inflammation, vascular changes, and oxidative stress. While research has focused on “pure AD,” most cases involve concomitant pathologies, such as α‐synuclein (dementia with Lewy bodies – DLB), TDP‐43 (Limbic‐predominant age‐related TDP‐43 encephalopathy ‐ LATE), and neuroinflammation, which can exacerbate amyloid and tau pathologies. While biomarkers for amyloid and tau are established, developing cross‐matrix biomarkers (tissue, CSF, and blood) of other pathologies is crucial for understanding trajectory of disease progression and patient heterogeneity. The Oxford Project to Investigate Memory and Ageing (OPTIMA) cohort was a longitudinal study of cognitively impaired and unimpaired participants established in Oxford in 1988. In this project, our aim is to integrate high throughput brain pathology with biofluid assays and proteomics to generate novel biomarkers for stratifying patients with AD and mixed pathologies.

**Method:**

The project will analyse selected cases from the OPTIMA cohort (*n* = 332), that include cases with pure AD, AD+DLB and/or LATE and healthy controls. Here we present our data from characterisation of baseline lumbar CSF (Amyloid, Tau and Neurodegeneration ‐ ATN). We determined pTau181, t‐Tau, Aβ42 and Aβ‐40 concentrations using EuroImmun ELISAs. Human samples were collected under appropriate ethics approval and with informed consent, and sourced from the Oxford Brain Bank under a Research Ethics Committee approved protocol (ref 23/SC/0241).

**Result:**

Here we present the characterisation of ATN status of CSF samples from the OPTIMA cohort. In addition, we include some data from post‐mortem ventricular CSF from a small subset (*n* = 4) of OPTIMA cases, which showed pTau181 levels were much higher in comparison to lumber CSF concentrations whereas Aβ‐42 levels were undetectable.

**Conclusion:**

The OPTIMA cohort continues to be an invaluable resource for advancing diagnostic and treatment approaches in neurodegenerative diseases. In addition, given the substantial concentration differences of ATN‐defining proteins in post‐mortem ventricular samples than in lumbar CSF taken during life, this may limit the utility of post‐mortem CSF as technical controls.

